# Causes and Management of Non-cirrhotic Portal Hypertension​

**DOI:** 10.1007/s11894-020-00792-0

**Published:** 2020-09-17

**Authors:** Stefania Gioia, Silvia Nardelli, Lorenzo Ridola, Oliviero Riggio

**Affiliations:** Department of Translational and Precision Medicine, Sapienza University of Rome, Rome, Italy

**Keywords:** Porto-sinusoidal vascular liver disease, Portal vein thrombosis, Portal hypertension

## Abstract

**Purpose of the Review:**

Non-cirrhotic portal hypertension (NCPH) includes a heterogeneous group of conditions. The aim of this paper is to make an overview on the denominations, diagnostical features and management of porto-sinusoidal vascular disease (PSVD) and chronic portal vein thrombosis (PVT) being the main causes of NCPH in the Western world.

**Recent Findings:**

The management of NCPH consists in the treatment of associated diseases and of portal hypertension (PH). PH due to PSVD or PVT is managed similarly to PH due to cirrhosis. TIPS placement and liver transplantation are considerable options in patients with refractory variceal bleeding/ascites and with progressive liver failure. Anticoagulation is a cornerstone both in the treatment of thrombosis in PSVD and in the prevention of thrombosis recurrence in patients with portal cavernoma.

**Summary:**

Physicians should be aware of the existence of PSVD and chronic PVT and actively search them in particular settings. To now, the management of portal hypertension-related complications in NCPH is the same of those of cirrhosis. Large cooperative studies on the natural history of NCPH are necessary to better define its management.

## Introduction

The term non-cirrhotic portal hypertension (NCPH) refers to a heterogeneous group of liver disorders that primarily affect the liver vascular system and that are classified anatomically on the basis of site of resistance to blood flow, as pre-hepatic, hepatic (pre-sinusoidal, sinusoidal or post-sinusoidal) and post-hepatic [1, 2]. The causes of NCPH are listed Table 1. For years they have been considered quite rare conditions, but their scarce frequency was mostly due to the poor knowledge and to the low grade of suspicion. However, in the last years, the multicentric cooperation together with the unification of the several terminologies and nomenclatures used to name these diseases led to deepen the knowledge on the clinical features, presentation and management of vascular liver diseases.

**Table 1:** Principal causes of non-cirrhotic portal hypertension (NCPH)

Pre-sinusoidal	Sinusoidal	Post-sinusoidal
Porto-sinusoidal vascular disease (PSVD)	Drug-induced	Budd-Chiari syndrome
Portal vein obstruction (neoplastic and non-neoplastic)	Alcoholic liver damage	Veno-occlusive disease
Schistosomiasis	Non-alcoholic steatohepatitis	Primary vascular malignancies
Arteriovenous fistulas	Viral hepatitis	Hypervitaminosis A
Polycystic disease	Amyloidosis	Epithelioid hemangio-endothelioma and angiosarcoma
Congenital hepatic fibrosis	Infiltrative diseases	
Biliary diseases (primary biliary cirrhosis; primary sclerosing cholangitis)	Acute fatty liver of pregnancy	
	Gaucher’s disease	
	Visceral leishmaniasis	

The present paper represents an overview on the denominations, diagnostical features and management of porto-sinusoidal vascular disease (PSVD) and chronic portal vein thrombosis (PVT) that represent the most frequent causes of NCPH in the Western world.

### Porto-Sinusoidal Vascular Disease (PSVD)

#### Definition and Nomenclature

The term porto-sinusoidal vascular disease (PSVD) regroups several conditions characterized by alterations of the small branches of portal veins previously named from an histological point of view as “obliterative portal venopathy”, “nodular regenerative hyperplasia”, “hepato-portal sclerosis”, “non-cirrhotic portal fibrosis” and “incomplete septal fibrosis” or from a clinical point of view as “idiopathic portal hypertension” and “idiopathic non-cirrhotic portal hypertension (INCPH)” [3•].

Almost recently, new diagnostic criteria have been proposed by the European Association for the Vascular Liver Disease (VALDIG) [4••] that defines the diagnosis of PSVD in the presence of one of the three following features:

i) Absence of cirrhosis at an adequate liver biopsy and at least one specific sign of portal hypertension
ii) Absence of cirrhosis at an adequate liver biopsy and at least one specific histological sign of PSVD
iii) Absence of cirrhosis at an adequate liver biopsy and at least one non-specific sign of portal hypertension and at least one non-specific histological sign of PSVD

Specific and non-specific clinical and histological signs are reported in Table 2.

**Table 2:** Diagnostical criteria for PSVD (VALDIG) [4••]

**Specific clinical signs of PH**	**Specific histological signs of PSVD**
Gastric-oesophagael, or ectopic varices Portal hypertensive bleeding Porto-systemic collaterals	Obliterative portal venopathy (thickening of vessel wall, occlusion of the lumen and vanishing of portal veins) Nodular regenerative hyperplasia Incomplete septal fibrosis or incomplete septal cirrhosis
**Unspecific clinical signs of PH**	**Unspecific histological signs of PSVD**
Ascites Spleen size ≥ 13 cm in the largest axis Platelet count < 150,000 per μL	Portal tract abnormalities (multiplication, dilation of arteries, periportal vascular channels and aberrant vessels) Architectural disturbance: irregular distribution of the portal tracts and central veins Non-zonal sinusoidal dilation Mild perisinusoidal fibrosis

The term PSVD replaced the term INCPH in order to include the patients with specific histological features but no clinical signs portal hypertension.

#### When to Suspect PSVD?

There are two clinical scenarios in whom PSVD can be suspected: patients with chronic and unexplained alteration of liver enzymes without portal hypertension and patients with unexplained clinically evident portal hypertension. In both the cases, known causes of chronic liver disease or portal hypertension should be excluded.

The alterations of liver tests are various, and in particular they are represented by a mild elevation of ALT and AST, by an elevation of alkaline phosphatase 2 times upper the normal value or by an elevation of gamma-GT. These alterations are not associated to signs of portal hypertension [5, 6].

Two recent European series showed that the histological lesions usually observed in patients affected by PSVD with portal hypertension (i.e. obliterative portal venopathy, OPV) were present in the 19% [7] and 25% [5] of the liver biopsies of patients with chronic elevation liver enzymes without cirrhosis and portal hypertension. Some of these patients developed clinical signs of portal hypertension during the follow-up. These observations suggest first of all that PSVD should be suspected and actively searched among the patients with chronic liver test abnormalities of unknown aetiology and no signs of portal hypertension and that, such conditions, PSVD with and without portal hypertension may be different stages of the same disease where histological PSVD might represent an “early” pre-symptomatic stage of PSVD with portal hypertension.

PSVD should be also suspected in patients with unexplained portal hypertension.

The absence of a cause of chronic liver disease together with the presence of a marked portal hypertension with normal or only mildly altered liver function tests should raise the suspicion of PSVD. If transaminases or cholestasis enzymes could sometimes be elevated, the liver synthetic capacity is usually preserved [8••]. In fact, in these patients, bilirubin and albumin are quite normal, and prothrombin time is usually superior up to 50%. Moreover, other laboratory alterations such as anaemia, leukopenia and thrombocytopenia are the consequences of hypersplenism. On this basis, the distinction between PSVD and cryptogenic compensated cirrhosis may be very difficult and should always be supported by a liver biopsy. However, liver elastography may be helpful in this differential diagnosis at least to suspect PSVD and to select patients to be submitted to liver biopsy. In fact, the presence of a low liver stiffness (< 10 kPa) in patients with clinically evident portal hypertension may make the diagnosis of cirrhosis unlikely [9].

Another challenge for the diagnosis of PSVD is with patients with chronic PVT. In fact, with new insights in the natural history of PSVD and its physiopathology, it is known that PSVD is frequently complicated by extrahepatic portal vein thrombosis [6, 8••], and, hypothetically, if a certain patient is investigated after PVT occurs, it may be impossible to determine if a pre-existent PSVD is the cause of the vascular disease. In these cases, the only way to investigate PSVD might be performing a liver biopsy. Hence, the presence of a pre-existing, undiagnosed PSVD should be suspected in patients with acute or chronic PVT, and that is why, the criteria of the patency of the portal vein has been eliminated in the last definition of PSVD.

Moreover, PSVD is frequently associated with several systemic conditions and with the chronic exposition to various drugs and toxins (Table 3) that may play a direct role in the pathophysiology of the liver alterations. It has been reported that more than a half of PSVD patients have an associated disease [2, 6, 10]. As a practical consequence, in patients affected by these diseases or exposed to these drugs, the searching of signs of portal hypertension is suggested. In particular, physicians should be aware of the possibility to develop PSVD, and they should be careful about the presence of liver tests alteration, of indirect signs of portal hypertension such as thrombocytopenia and of splenomegaly or portal vein dilatation, when an imaging technique is performed. When one of these alterations is present, the patient should be referred to a hepatologist.

**Table 3:** Diseases and drugs associated to porto-sinusoidal vascular disease (PSVD)

**Thrombophilia**	**Hematologic disease**
Protein S, protein C, antithrombin deficiency Antiphospholipid syndrome Factor V Leiden Prothrombin mutation	Myeloproliferative Neoplasm Myeloid metaplasia Lymphoproliferative disorders (Hodgkin’s disease, non-Hodgkin’s lymphoma, chronic lymphocytic leukaemia and multiple myeloma) Spherocytosis
**Genetic disorders**	**Gut Diseases**
Cystic fibrosis Adams Oliver syndrome Turner’s disease TERT/TERC mutation	Celiac disease Inflammatory bowel disease
**Autoimmune disease**	**Acquired and congenital immunodeficiency**
Rheumatoid arthritis Systemic lupus erythematosus Systemic sclerosis Scleroderma	HIV infection Primary antibody-deficiency syndrome
**Drug and toxics**	
Oxaliplatin Azathioprine, 6-thioguanine Cytosine arabinoside Cyclophosphamide Bleomycin Chlorambucil Doxyrubicin Carmustine	

#### Diagnosis

For the diagnosis of PSVD, a combination of histological and radiological findings is necessary.

Biopsy remains mandatory to confirm the diagnosis of PSVD. Since the typical histological lesions cannot be all always contemporary present and they are distributed in a focal way, it is necessary to have an adequate liver specimen (Table 2) [4••, 11•]. The optimal sample should measure more than 20 mm of length and contain at least 10 portal spaces, and it should be low fragmented. It is equally important that the liver biopsy would be referred to an expert pathologist.

Unfortunately, there are not specific radiological signs of PSVD, but the combination of several findings may suggest the diagnosis of PSVD.

Doppler ultrasound is frequently the first examination performed in patients with suspicion of PSVD. In these patients, the liver aspect may be either normal or inhomogeneous with irregular surface due to the micronodular transformation, rendering very difficult to differentiate it from cirrhosis, and caudal lobe hypertrophy and right hepatic lobe atrophy are present. In PSVD the main findings are the signs of portal hypertension such as splenomegaly, even more marked than in patients with cirrhosis, and portal venous axis dilatation. Moreover, in PSVD, the portal vein may appear markedly thickened with hyperechoic walls, anomalies that are found also in its intrahepatic branches, probably indicating periportal fibrosis.

Computed tomography (CT) would reveal vascular abnormalities especially on the peripheral intrahepatic portal branches (i.e. heterogeneous hepatic enhancement, abrupt narrowing of second-degree intrahepatic portal vein branches, paucity of the medium size portal branches). Moreover, CT has a better performance than Doppler ultrasound once a portal vein thrombosis is found, in order to assess its extension and duration but also for the evaluation of porto-systemic shunts. Finally, CT may be helpful to assess the presence of benign hypervascular nodules due to the haemodynamic abnormalities [12, 13].

Liver stiffness may have a role in the diagnosis of PSVD at least to rule out the presence of cirrhosis. In these patients, liver stiffness can be normal or slightly elevated but surely lower than cirrhosis [9]. The presence of clinically relevant portal hypertension with normal or moderate elevated values of liver stiffness should lead to exclude cirrhosis and to suspect PSVD. Furthermore, liver stiffness measurement would be helpful also in the distinction between patients with PVT caused and not caused by PSVD being it higher in patients with PVT secondary to PSVD [14, 15].

Owing to the fact that patients with PSVD have a pre-sinusoidal type of portal hypertension, the HVPG is normal or slightly elevated, frequently < 10 mmHg [9, 16, 17]. Moreover, the presence of vein-to-vein communications, often seen in these patients with a frequency higher than in cirrhotic patients, may further underestimate the value of HVPG. Hence, contrarily to cirrhosis, haemodynamic studies have scarce utility to indirectly evaluate the severity of portal hypertension in patients with PSVD where the HVPG is not correlated with clinical events such as the development of oesophageal varices, variceal bleeding or ascites.

### Chronic Portal Vein Thrombosis (PVT)

#### Definition and Nomenclature

Portal vein thrombosis refers to a primary obstruction by a thrombus located on the trunk or the left or right branches of the portal vein in the absence of malignant invasion or constriction. The adjective “chronic” refers to a long-standing thrombosis, and the term “portal cavernoma” (or cavernomatous transformation of the portal vein) defines the set of collateral veins replacing the portal vein. In adult patients, these two terms are synonymous, while in children, portal cavernoma may be the consequence of a congenital malformation as well as the sequel of PVT [18–20].

#### Aetiology

In 75% of patients with PVT and without an underlying liver disease (cirrhosis or PSVD), a risk factor for venous thrombosis is identified. Risk factors are various, and they are divided into local and systemic [21]. Local risk factors are mainly represented by inflammatory conditions affecting intraperitoneal organs, and they can be found in only one-third of the patients. Systemic risk factors are more frequent, and they are represented especially by thrombophilic conditions (i.e. protein S or C deficiency, antiphospholipid antibodies, factor V Leiden and prothrombin mutation) and myeloproliferative neoplasm. However, in 25% of patients, no aetiological factors for the thrombosis are identified despite an active search.

#### Diagnosis

The diagnosis of chronic PVT is based on the findings of Doppler ultrasound and axial CT or MR imaging using vascular contrast agents. Diagnosis is based on the absence of blood flow into portal vein and on the presence of numerous, serpiginous vascular channels in porta hepatis corresponding to portal cavernoma [22–24]. Once a portal cavernoma is identified at Doppler ultrasound, a CT scan or MR should be performed to more accurately define the extension of the thrombosis and the identification of signs of portal hypertension. Moreover, other less specific features of the disease are the presence of a dysmorphic liver where segment 1 and segment 4 are enlarged but surface is smooth; a mosaic pattern of parenchymal enhancement in the arterial phase, with homogeneous enhancement at a later phase; an increased enhancement of the peripheral parts of the liver at the arterial phase; a dilated hepatic artery; and a mild irregular dilatation of intra- and extrahepatic bile secondary to the compression by the collateral veins constituting the cavernoma (portal biliopathy). MR imaging cholangiography is the choice imaging for the diagnosis of portal biliopathy [25]. As further consequence, both the gallbladder and pancreas may appear with thickened and heterogenous wall that should be differentiated from cholecystitis and pancreatic cancer and chronic pancreatitis, respectively.

Laboratory findings include a mild or absent liver dysfunction with normal levels of transaminases. These findings typically contrast with the severity of signs of portal hypertension. Alkaline phosphatase and gamma-glutamyl transferase may be altered in the presence of portal biliopathy.

In cases of pure portal vein thrombosis, liver biopsy shows an essentially normal liver, and it is actually not indicated unless in the presence of persistently abnormal liver tests, of a dysmorphic liver or of abnormal results of liver elastometry in the suspicion of an underlying liver disease (cirrhosis and PSVD) [18, 26, 27].

Non-invasive tests like elastometry would be most useful in recognizing underlying liver disease [9, 15, 28].

## Natural History and Management

### General Management

Both in patients with PSVD and chronic PVT, the outcome is mostly determined by age and the course of the underlying disease. Therefore, the first step for the management of these patients is the early detection and treatment of the diseases known to be associated to PSVD and PVT (i.e. HIV infection, immunodeficiency, haematological disorders).

Natural history of PSVD without portal hypertension is not known [4••]. Probably a proportion of the patients with histological lesions will never develop portal hypertension. However, as previously exposed, it has been reported that a percentage of patients with histological PSVD developed signs of portal hypertension during follow-up [5]. Hence, to date, it is not clear what is the appropriate follow-up and management of this kind of patients. Probably, a clinical, radiological and endoscopic follow-up is considerable at least to early detect the development of signs of portal hypertension.

Natural history of patients with portal hypertension due to PSVD or chronic PVT is better known, and it is mainly characterized by the complications of portal hypertension itself.

### Oesophageal Varices and Variceal Bleeding

The main complication of the diseases is the gastrointestinal bleeding from the rupture of oesophago-gastric varices. This complication is highly frequent both in patients receiving and not receiving a treatment for the prophylaxis of variceal bleeding. It has been reported [8••] that the incidence of variceal bleeding as well as the rate of development of varices at risk of bleeding is higher than in patients with cirrhosis independently on the size of varices at the first endoscopy. The observation of a more rapidly progression of varices in this kind of patients should probably suggest the necessity of a different timing of the endoscopical follow-up, maybe closer than in cirrhosis. Nevertheless, in the absence of large prospective and cooperative studies in patients with non-cirrhotic portal hypertension, current guidelines on vascular liver diseases suggest to manage this complication according to the guidelines of cirrhotic portal hypertension [18, 20].

Regarding the type of prophylaxis, one study compared endoscopic variceal ligation versus endoscopic variceal ligation plus non-selective beta-blockers for the primary prophylaxis [29], and two studies did the same comparison in the setting of secondary prophylaxis for variceal bleeding [29, 30] in patients with PSVD. All these studies did not provide solid conclusions due to the very small number of patients included. Therefore, by extrapolating the recommendations for cirrhosis, non-specific beta-adrenergic blockade or endoscopic band ligation is used for primary prophylaxis and their combination for secondary prophylaxis in patients with NCPH [18–20].

Transjugular intrahepatic porto-systemic shunt (TIPS) is to consider a valid option for the patients with variceal bleeding not controlled by medical and endoscopical treatment. In this setting, the results are favourable in patients with PSVD [31]. In fact, an international multicenter retrospective study showed an 80% 2-year survival in patients with PSVD treated with TIPS [32]. Also, in patients with chronic PVT, TIPS is a considerable option but not always feasible due to the technical challenge of inserting a vascular prosthesis into the cavernomatous veins which may compromise medium-term patency and efficacy of the stent [33, 34]. Another possible indication to the use of TIPS is to allow a lifelong anticoagulation therapy in selected patients with chronic PVT and high-risk varices. However, these patients should be referred to selected units with large experience in TIPS placement.

Finally, mortality due to variceal bleeding is significantly lower than that observed in cirrhotic patients, likely because of a preserved liver function, about 3% at 6 weeks [6, 8••, 35].

### Ascites, Hepatic Encephalopathy and Other Complications

Ascites is not a frequent complication of PSVD, and it usually occurs during decompensating events such as infections or variceal bleeding. In patients with chronic PVT, ascites is probably even more infrequent and may develop with increasing age, prolonged duration of disease and development of portal biliopathy, and such patients generally have reduced hepatic cell mass and synthetic dysfunction [36].

Indeed, in the absence of solid studies, the management of ascites is the same of cirrhotic patients. TIPS placement represents an option for PSVD patients with refractory ascites. In this setting, the outcomes may be less favourable than in patients with variceal bleeding because the presence of severe comorbidities and of renal impairment (creatinine ≥ 1.13 mg/dL) has been associated with a poor survival after TIPS [32].

Hepatic encephalopathy, both overt and minimal, is a complication much less frequent than in cirrhosis, but it has been reported to occur in 32% of patients with NCPH [37, 38]. The development of HE in these patients is strictly related to the presence of large porto-systemic shunt either spontaneous or iatrogenic (type B HE). In fact, in the series of PSVD patients undergoing to TIPS, the 31% of patients developed this condition following the stent’s placement. In most cases, it was transient and easily managed with medical treatment [32].

Contrarily to cirrhosis, the risk of developing a hepatocellular carcinoma is very low, and only two cases have been reported to date in patients with NCPH. On the contrary, hypervascular benign lesions sharing the features of FNH-like lesions were seen in 14% of patients with PSVD and not in patients with cirrhosis in a French’ series [12], and they are a common finding also in patients with chronic PVT [39]. These lesions are the consequence of the haemodynamic alterations proper to the disorders, and when they are observed in PSVD patients with morphologic liver changes similar to those of cirrhosis, they are very difficult to distinguish from hepatocellular carcinoma.

Moreover a recent study [40] reported that in patients with NCPH, the prevalence of sarcopenia, a common complication of cirrhosis, studied by CT scan, was similar (36%) to those observed in cirrhotic patients and that it had a clinical negative impact on the natural history of the disease being the main predictor of refractory variceal bleeding independently on well-known predictors of portal hypertension severity such as the variceal size and use of beta blockers.

Finally, the evolution to progressive liver failure is rare but possible, and it represents an indication to liver transplantation in patients with PSVD together with the presence of intractable complications of portal hypertension [35, 41]. The number of reported transplants in patients with PSVD is very low and includes patients diagnosed after evaluating the explant in patients with presumed cryptogenic cirrhosis [41]. There are reported outcome recurrences of PSVD after liver transplantation [42, 43].

Overall outcome is relatively good both in patients with NCPH. Five-year survival rates above 70% have been reported in large cohorts spanning over the last 20 years for patients with chronic PVT [44–46], and 10-year survival rates above 56–82% have been reported for patients with PSVD [35].

### Portal Vein Thrombosis and Anticoagulants

#### Porto-Sinusoidal Vascular Disease (PSVD)

The other main complication of PSVD is represented by extrahepatic portal vein thrombosis. This event occurs in 30–40% of patients, with an incidence much higher than in patient with cirrhosis [6, 8••]. This higher incidence is sustained by the strong association between PSVD, prothrombotic conditions and the slowing down of the blood flow in the portal vein axis secondary to portal hypertension.

For an early detection of portal vein thrombosis, current guidelines [18] suggest to perform Doppler ultrasonography every 6 months even if no literature supporting this practice exists until now.

Nevertheless, a prophylactic treatment with anticoagulants is not recommended at this time, and long-term anticoagulation should be considered when an underlying prothrombotic disorder is diagnosed, and it is strongly suggested in patients who develop portal vein thrombosis [18, 20] even if no studies demonstrated a clinical negative impact of PVT on the natural history of the disease. However, in these cases, anticoagulation should be carried on long life because re-thrombosis is frequent at the withdrawal of the therapy.

Furthermore, in the future, it would be interesting to evaluate if the use of anticoagulants may play a role in the prevention of the progression to clinically evident portal hypertension in patients with histological signs of PSVD without portal hypertension.

#### Chronic Portal Vein Thrombosis (PVT)

In patients with portal cavernoma, the aim of anticoagulant treatment is not to achieve the recanalization of the portal vein axis as in acute portal vein thrombosis, but it is represented by the prevention of the thrombotic extension and recurrence in the splanchnic area. However, the indications for permanent anticoagulation are still unclear. The discussion on a case-by-case basis should take into account the thrombotic potential of the underlying conditions and the extension of the thrombus to the superior mesenteric vein [47, 48]. In fact, at present, patients with cavernoma with the poorest outcomes usually have involvement of the superior mesenteric and splenic veins.

Therefore, current guidelines suggest to consider long-term anticoagulation surely in the presence of persistent prothrombotic conditions and in patients with history of intestinal ischemia or abdominal pain. Uncertainty remains on the indication of anticoagulation in patients without strong prothrombotic conditions. The indication to the therapy should also take into consideration the risk-benefit ratio of anticoagulation in patients with portal hypertension even if it has been reported that the risk of bleeding is not increased in patients under anticoagulants when an adequate prophylaxis for bleeding is performed [44–46]. Moreover, the severity of bleeding is not increased by anticoagulation [42] that, on the contrary, seems to have a favourable impact on survival in these patients [49].

Regarding the type of anticoagulation for both patients with chronic PVT and PSVD, the most commonly used are heparin (un-fractioned heparin (UFH) and low-molecular-weight heparin (LMWH)) and vitamin K antagonist (VKA). There are few data on the use of direct oral anticoagulants (DOACs) in patients with PSVD and non-cirrhotic portal vein thrombosis. The use of DOACs (rivaroxaban, apixaban and dabigatran) has been evaluated in splanchnic vein thrombosis, but no solid conclusions can be made especially on the type and dosage of the agent and on the adverse events’ monitoring/management. Based on the limited data, it is safe to only conclude that the use of DOACs in patients with portal hypertension is feasible and that it does not increase the haemorrhagic risk, but it should remain an individual option [50•, 51, 52].

## Abbreviations

PSVD: Porto-sinusoidal vascular liver disease
PVT: Portal vein thrombosis
NCPH: Non-cirrhotic portal hypertension
INCPH: Idiopathic non-cirrhotic portal hypertension
OPV: Obliterative portal venopathy
LS: Liver stiffness
CT: Computed tomography
TIPS: Transjugular intrahepatic porto-systemic shunt
